# Supplementary material for: Contamination and Health Risk Assessment of Polycyclic Aromatic Hydrocarbons in Seasoning Flour Products in Hunan, China
Source: Int J Environ Res Public Health. 2023 Jan 5;20(2):963. doi: 10.3390/ijerph20020963 (PMC9859540; doi:10.3390/ijerph20020963)
Supplement: Supplementary file 1 [file ijerph-20-00963-s001.zip › ijerph-2126415-supplementary.pdf]

Supplementary materials

Table S1. Contamination of 16 PAHs congeners in various seasoning flour products (LB).

PAHs	Taste														
	Soggy (n = 51)					Crisp (n = 12)					Chewy (n = 16)				
	>LOD (%)	P_{25} (μg/kg)	Median (μg/kg)	P_{75} (μg/kg)	Maximum (μg/kg)	>LOD (%)	P_{25} (μg/kg)	Median (μg/kg)	P_{75} (μg/kg)	Maximum (μg/kg)	>LOD (%)	P_{25} (μg/kg)	Median (μg/kg)	P_{75} (μg/kg)	Maximum (μg/kg)
NAP	45.10	0.000	0.000	3.076	41.347	66.67	0.000	2.992	6.603	77.677	81.25	0.289	1.667	15.346	21.137
ACY	29.41	0.000	0.000	0.742	19.466	58.33	0.000	3.180	5.626	24.093	68.75	0.000	0.798	2.404	14.141
ACE	31.37	0.000	0.000	0.668	5.460	83.33	0.831	4.099	7.558	9.835	93.75	0.568	1.433	3.727	7.913
FLU	49.02	0.000	0.000	3.570	20.167	83.33	0.809	1.792	3.566	10.627	87.50	0.633	6.178	8.909	16.746
PHE	52.94	0.000	0.797	9.082	58.961	83.33	0.519	2.197	3.988	29.473	93.75	2.088	13.062	29.181	43.975
ANT	37.25	0.000	0.000	1.261	6.675	58.33	0.000	1.704	4.431	5.986	37.50	0.000	0.000	2.709	4.816
FLA	52.94	0.000	0.954	7.390	26.471	91.67	0.600	3.819	5.332	17.919	93.75	2.059	8.662	15.340	20.985
PYR	50.98	0.000	0.307	2.582	13.158	83.33	0.614	3.152	4.730	9.052	93.75	2.525	4.483	7.559	9.792
BaA	72.55	0.000	1.599	3.572	10.178	91.67	1.980	2.890	3.783	6.494	100.00	1.780	4.464	6.010	6.879
CHR	66.67	0.000	1.107	3.865	10.600	91.67	2.234	3.305	4.485	7.101	93.75	1.913	4.182	6.552	7.959
BbF	21.57	0.000	0.000	0.000	2.878	50.00	0.000	0.273	3.256	4.387	37.50	0.000	0.000	1.785	2.599
BkF	33.33	0.000	0.000	2.157	5.613	83.33	2.029	2.776	3.817	4.875	50.00	0.000	0.178	2.424	6.484
BaP	50.98	0.000	0.348	1.028	8.184	91.67	0.770	1.534	4.024	6.214	93.75	0.902	1.209	2.274	4.917
IcdP	37.25	0.000	0.000	0.463	3.656	75.00	0.118	0.696	2.381	3.997	87.50	0.353	0.482	0.838	1.819
DahA	11.76	0.000	0.000	0.000	3.135	41.67	0.000	0.000	3.033	4.243	25.00	0.000	0.000	0.266	2.165
BghiP	37.25	0.000	0.000	0.322	2.272	50.00	0.000	0.276	0.714	1.602	50.00	0.000	0.178	0.498	0.760

PAHs	Filamentous (n = 11)					Rod-shaped (n = 35)					Flaky (n = 25)					Granular (n = 8)				
	>LOD (%)	P_{25} (μg/kg)	Median (μg/kg)	P_{75} (μg/kg)	Maximum (μg/kg)	>LOD (%)	P_{25} (μg/kg)	Median (μg/kg)	P_{75} (μg/kg)	Maximum (μg/kg)	>LOD (%)	P_{25} (μg/kg)	Median (μg/kg)	P_{75} (μg/kg)	Maximum (μg/kg)	>LOD (%)	P_{25} (μg/kg)	Median (μg/kg)	P_{75} (μg/kg)	Maximum (μg/kg)
NAP	18.18	0.000	0.000	0.000	12.680	77.14	0.848	2.917	13.208	77.677	56.00	0.000	0.426	3.939	23.609	12.50	0.000	0.000	0.000	0.303
ACY	27.27	0.000	0.000	0.782	4.173	48.57	0.000	0.000	2.633	24.093	52.00	0.000	0.360	2.716	21.451	0.00	0.000	0.000	0.000	0.000
ACE	9.09	0.000	0.000	0.000	5.132	62.86	0.000	0.853	3.203	8.248	68.00	0.000	0.623	4.681	9.835	12.50	0.000	0.000	0.000	0.582
FLU	9.09	0.000	0.000	0.000	3.204	80.00	0.601	2.201	6.798	20.167	76.00	0.296	2.363	6.729	11.383	12.50	0.000	0.000	0.000	5.065
PHE	18.18	0.000	0.000	0.000	2.638	82.86	0.971	3.900	14.521	58.961	80.00	0.560	3.779	19.774	32.329	12.50	0.000	0.000	0.000	13.572
ANT	36.36	0.000	0.000	1.774	6.675	48.57	0.000	0.000	1.952	5.986	44.00	0.000	0.000	2.430	4.851	0.00	0.000	0.000	0.000	0.000
FLA	27.27	0.000	0.000	0.954	2.593	80.00	0.171	5.135	7.921	26.471	80.00	1.578	5.246	13.140	21.070	25.00	0.000	0.000	3.056	10.666
PYR	9.09	0.000	0.000	0.000	2.818	77.14	0.186	2.154	4.102	13.158	84.00	1.607	3.836	6.641	12.208	25.00	0.000	0.000	0.800	5.874
BaA	54.55	0.000	0.278	1.333	2.657	91.43	1.711	3.006	3.916	8.611	92.00	1.856	3.572	5.674	10.178	37.50	0.000	0.000	2.164	4.537
CHR	36.36	0.000	0.000	0.802	5.000	91.43	1.773	3.289	4.612	10.600	88.00	2.047	3.538	5.768	9.885	25.00	0.000	0.000	1.562	4.318
BbF	18.18	0.000	0.000	0.000	1.699	34.29	0.000	0.000	0.703	4.146	36.00	0.000	0.000	1.420	4.387	0.00	0.000	0.000	0.000	0.000
BkF	36.36	0.000	0.000	2.854	5.432	42.86	0.000	0.000	2.480	6.434	56.00	0.000	1.357	3.399	4.875	25.00	0.000	0.000	0.932	4.108
BaP	27.27	0.000	0.000	1.953	8.184	77.14	0.348	0.749	1.307	7.728	80.00	0.713	1.179	2.763	4.485	25.00	0.000	0.000	0.529	1.412
IcdP	9.09	0.000	0.000	0.000	3.656	60.00	0.000	0.400	0.575	3.246	72.00	0.000	0.552	1.091	3.997	25.00	0.000	0.000	0.347	0.917
DahA	9.09	0.000	0.000	0.000	3.135	22.86	0.000	0.000	0.000	4.084	24.00	0.000	0.000	0.212	4.243	0.00	0.000	0.000	0.000	0.000
BghiP	9.09	0.000	0.000	0.000	1.501	42.86	0.000	0.000	0.446	1.602	60.00	0.000	0.299	0.554	2.272	25.00	0.000	0.000	0.228	0.794

Naphthalene (NAP), acenaphthylene (ACY), acenaphthene (ACE), fluorene (FLU), phenanthrene (PHE), anthracene (ANT), fluoranthene (FLA), pyrene (PYR), ben-zo[a]anthracene (BaA), chrysene (CHR), benzo[b]fluoranthene (BbF), benzo [k]fluoranthene (BkF), benzo[a]pyrene (BaP), indene[1,2,3-c,d]pyrene (IcdP), dibenz [a,h]anthracene (DahA), benzo[g,h,i]perylene (BghiP). >LOD (%): the proportion of content above the limit of detection.

Table S2. Concentrations of four PAHs pollution indicators in various seasoning flour products (LB).

Seasoning Flour Product	BaP		PAH2		PAH4		PAH8	
	Median (P25, P75) (μg/kg)	P_{95} (μg/kg)	Median (P25, P75) (μg/kg)	P_{95} (μg/kg)	Median (P25, P75) (μg/kg)	P_{95} (μg/kg)	Median (P25, P75) (μg/kg)	P_{95} (μg/kg)
**Taste**								
Soggy (*n* = 51)	0.348 (0.000, 1.028)	3.911	1.953 (0.000, 4.831)	12.347	3.067 (0.278, 8.747)	18.364	4.209 (1.317, 10.597）	24.483
Crisp (*n* = 12)	1.534 (0.770, 4.024)	5.263	6.032 (3.008, 8.238)	9.735	11.140 (5.189, 15.775)	16.122	15.613 (7.438, 22.103)	28.86
Chewy (*n* = 16)	1.209 (0.902, 2.274)	3.458	5.411 (4.212, 7.576)	8.72	10.107 (7.664, 13.578)	15.989	13.195 (10.330, 15.916)	19.186
**Shape**								
Filamentous (*n* = 11)	0.000 (0.000, 1.953)	5.077	0.339 (0.000, 1,953)	7.577	1.288 (0.000, 2.234)	10.304	2.234 (1.288, 3.132)	17.692
Rod-shaped (*n* = 35)	0.749 (0.348, 1.307)	5.306	4.094 (1.982, 6.704)	11.285	7.629 (4.209, 11.554)	18.305	8.180 (4.793, 13.768)	26.36
Flaky (*n* = 25)	1.179 (0.713, 2.763)	4.077	6.477 (3.986, 7.284)	8.751	10.482 (7.480, 13.633)	15.965	13.573 (8.150, 18.260)	24.584
Granular (*n* = 8)	0.000 (0.000, 0.529)	1.165	0.000 (0.000, 2.090)	4.7	0.000 (0.000, 4.255)	8.609	0.216 (0.000, 8.113)	11.427
**Total** (*N* = 79)	0.798 (0.000, 1.689)	4.528	3.998 (0.368, 6.704)	11.008	7.406 (1.594, 12.303)	17.579	8.180 (2.301, 14.283)	25.784

BaP, PAH2 (BaP + CHR), PAH4 (BaP + CHR+ BaA+ BbF), and PAH8 (BaP + CHR + BaA + BbF + BkF + DahA + BghiP + IcdP).

Table S3. Daily dietary exposure and the margin of exposure of PAHs (LB).

	Pollutant	$BMDL_{10}$	EDI		MOE	
		[mg/(kg·BW)/d]	P_{50} [ng/(kg·BW)/d]	P_{95} [ng/(kg·BW)/d]	P_{50}	P_{95}
Seasoning flour product						
Soggy	BaP	0.133	1.498	0.07	525194	46732
	PAH2	0.748	4.729	0.17	227273	35949
	PAH4	1.175	7.033	0.34	289445	48341
	PAH8	1.612	9.377	0.49	303961	52256
Crisp	BaP	0.245	0.842	0.07	285202	83127
	PAH2	0.965	1.558	0.17	176144	109142
	PAH4	1.782	2.580	0.34	190754	131807
	PAH8	2.498	4.618	0.49	196151	106116
Chewy	BaP	0.214	0.612	0.07	327114	114367
	PAH2	0.958	1.543	0.17	177500	110144
	PAH4	1.789	2.830	0.34	190057	120139
	PAH8	2.336	3.396	0.49	209804	144291
Population group						
Children	BaP	0.342	1.943	0.07	204474	36036
	PAH2	1.715	4.722	0.17	99117	35998
	PAH4	3.177	7.541	0.34	107013	45085
	PAH8	3.509	11.061	0.49	139632	44298
Adolescent	BaP	0.626	3.554	0.07	111744	19693
	PAH2	3.138	8.641	0.17	54167	19673
	PAH4	5.814	13.800	0.34	58482	24639
	PAH8	6.421	20.240	0.49	76309	24209
Adult	BaP	0.585	3.319	0.07	119672	21091
	PAH2	2.931	8.069	0.17	58010	21069
	PAH4	5.429	12.885	0.34	62631	26386
	PAH8	5.996	18.900	0.49	81722	25926

EDI, estimated daily intake; BMDL10, benchmark dose lower confidence limit; MOE, margin of exposure. The BMDL10 values were obtained from EFSA.

Table S4. The TEQBaP values of various seasoning flour products.

PAHs	TEF	UB							
		Soggy		Crisp		Chewy		Total	
		Median (μg/kg)	TEQ	Median (μg/kg)	TEQ	Median (μg/kg)	TEQ	Median (μg/kg)	TEQ
**Naphthalene (NAP)**	0.001	0.160	0.000	2.992	0.003	1.667	0.002	0.524	0.001
**Acenaphthylene (ACY)**	0.001	0.320	0.000	3.180	0.003	0.798	0.001	0.320	0.000
**Acenaphthene (ACE)**	0.001	0.280	0.000	4.099	0.004	1.433	0.001	0.346	0.000
**Fluorene (FLU)**	0.001	0.350	0.000	1.792	0.002	6.178	0.006	1.539	0.002
**Phenanthrene (PHE)**	0.001	0.797	0.001	2.197	0.002	13.062	0.013	2.047	0.002
**Anthracene (ANT)**	0.100	0.550	0.055	1.704	0.170	0.550	0.055	0.550	0.055
**Fluoranthene (FLA)**	0.001	0.954	0.001	3.819	0.004	8.662	0.009	2.769	0.003
**Pyrene (PYR)**	0.001	0.307	0.000	3.152	0.003	4.483	0.004	1.896	0.002
**Benzo[a]anthracene (BaA)**	0.100	1.599	0.160	2.889	0.289	4.464	0.446	2.480	0.248
**Chrysene (CHR)**	0.010	1.107	0.011	3.304	0.033	4.182	0.042	2.447	0.024
**Benzo[b]fluoranthene (BbF)**	0.100	0.210	0.021	0.378	0.038	0.210	0.021	0.000	0.000
**Benzo[k]fluoranthene (BkF)**	0.100	0.300	0.030	2.776	0.278	0.328	0.033	0.300	0.030
**Benzo[a]pyrene (BaP)**	1.000	0.348	0.348	1.534	1.534	1.209	1.209	0.798	0.798
**Indeno[1,2,3-c,d]pyrene (IcdP)**	5.000	0.330	1.650	0.696	3.480	0.482	2.410	0.379	1.895
**Dibenz[a,h]anthracene (DahA)**	0.010	0.240	0.002	0.240	0.002	0.240	0.002	0.240	0.002
**Benzo[g,h,i]perylene (BghiP)**	0.100	0.220	0.022	0.386	0.039	0.288	0.029	0.200	0.020
**Total**		8.072	2.303	35.138	5.884	48.236	4.284	16.835	3.082

		LB							
		**Soggy**		**Crisp**		**Chewy**		**Total**	
**PAHs**	**TEF**	**Median (μg/kg)**	**TEQ**	**Median (μg/kg)**	**TEQ**	**Median (μg/kg)**	**TEQ**	**Median (μg/kg)**	**TEQ**
NAP	0.001	0.000	0.000	2.992	0.003	1.667	0.002	0.524	0.001
ACY	0.001	0.000	0.000	3.180	0.003	0.798	0.001	0.000	0.000
ACE	0.001	0.000	0.000	4.099	0.004	1.433	0.001	0.346	0.000
FLU	0.001	0.000	0.000	1.792	0.002	6.178	0.006	1.539	0.002
PHE	0.001	0.797	0.001	2.197	0.002	13.062	0.013	2.047	0.002
ANT	0.1	0.000	0.000	1.704	0.170	0.000	0.000	0.000	0.000
FLA	0.001	0.954	0.001	3.819	0.004	8.662	0.009	2.769	0.003
PYR	0.001	0.307	0.000	3.152	0.003	4.483	0.004	1.896	0.002
BaA	0.1	1.599	0.160	2.890	0.289	4.464	0.446	2.480	0.248
CHR	0.01	1.107	0.011	3.305	0.033	4.182	0.042	2.447	0.024
BbF	0.1	0.000	0.000	0.273	0.027	0.000	0.000	0.000	0.000
BkF	0.1	0.000	0.000	2.776	0.278	0.178	0.018	0.000	0.000
BaP	1	0.348	0.348	1.534	1.534	1.209	1.209	0.798	0.798
IcdP	5	0.000	0.000	0.696	3.480	0.482	2.410	0.379	1.895
DahA	0.01	0.000	0.000	0.000	0.000	0.000	0.000	0.000	0.000
BghiP	0.1	0.000	0.000	0.276	0.028	0.178	0.018	0.000	0.000
Total		5.112	0.521	34.685	5.860	46.976	4.179	15.225	2.975

TEQ: the toxic equivalent concentration.

Table S5. The daily dietary exposure of TEQBaP (LB).

	Children	Adolescent	Adult
Soggy	5.204	19.272	21.116
Crisp	58.526	216.762	237.496
Chewy	41.737	154.581	169.367
Total	29.707	110.027	120.551

Table S6. The incremental lifetime cancer risk (LB).

	N	Mean	SD
Children	123	0.533	1.552
Adolescent	436	2.434	4.158
Adult	260	1.591	2.582
Total	819	1.881	3.483

No.				

Questionnaire on intake of seasoning flour products (spicy strip)

Hello! We are a joint project team of Central South University and Changsha County General Testing Centre. We are conducting a survey on the level of polycyclic aromatic hydrocarbons (PAHs) in food consumed by residents, so please take a few minutes to help fill out this questionnaire. The questionnaire is anonymous and the data obtained will be used for statistical analysis only. Thanks for your help!

Part 1. Basic Information

1. What is your gender? A.Male B. Female
2. Your current residence address is in ______ county (or township), ______ city (or district), ______ province.
3. Your birth date is in ______ (month)______ (year).
4. Your weight is ______ (kilograms).
5. Your height is ______(centimeters).
6. What is your usual preference for the flavor of food?
   A.Prefer spicy food.
   B.Prefer light food.
   C.No special preference, both spicy and light food are OK.
7. What is your educational background?
   A. Illiteracy.
   B. Primary school education.
   C. Middle and/or high school education.
   D. Junior college or bachelor degree.
   E. Master degree and higher.
8. What is your major?
   A.Medicine-related majors.
   B.Food-related majors.
   C.Others.
9. What is your occupation?
   A.Student.
   B. Employee of enterprises.
   C. Civil servant.
   D. Self-employed.
   E. Housewife (househusband)
   F. Retiree.
   G. Freelancer.
   H. Farmer.
   I. Others
10. How much is the per capita monthly household income of your family?
   A.1000 yuan or less. B. 1000-2000 yuan. C. 2000-3000 yuan. D. 3000-5000 yuan. E. 5000 yuan and more.
11. Do you suffer from underlying diseases (e.g. diabetes, cardiovascular diseases, hypertension, hyperlipidemia, tumors, etc.)?
   A.Yes B. No

12. What is your father's educational background?

A.Primary school education.

B. Junior middle school.

C. High school education.

D. Associate or bachelor degree and higher.

13. What is your mother's educational background?

A.Primary school education.

B. Junior middle school.

C. High school education.

D. Associate or bachelor degree and higher.

14. How about your parents' health status? Whether they suffer from underlying diseases (e.g. diabetes, cardiovascular diseases, hypertension, hyperlipidemia, tumors, etc.) or not?

A.Neither of them has underlying disease.

B.One has underlying disease and the other does not.

C.Both of them have underlying diseases.

Part 2. Food intake-related conditions

1. What is your attitude towards food safety?

A. Indifferent B. Less taken seriously C. Taken seriously D. Taken more seriously.

2. What do you think are common food safety problems (multiple choice)?

A. Expired and deteriorated food.

B. Products without date of manufacture, quality certification and information of manufacturer (Three no products).

C. Illegally added food additives or other chemical agents

D. Harmful substances generated or contaminated during food processing, production and transportation.

E. adulteration, adulteration, forgery

3. How about your eating habits?

A. Meat, eggs and milk are the mainstay in the diet, supplemented by vegetables and fruits.

B. The proportion of meat, eggs and milk and vegetables and fruits are roughly equivalent.

D. No fixed eating diet.

4. Are you aware of the harmful effects of PAHs on humans?

A. Understand B. Basically understand C. Don't understand

5. What is your attitude towards snacks?

A. Dislike B. Moderate C. Like D. Extremely like.

6. What do you think about the harmful impacts of spicy strips on health?

A. Almost none B. Average C. Large

7. What do you think of the quality of the oil used in the processing of spicy strips?

A. Very good B. Average C. Very poor

8. What is your average consumption of spicy strips?

A.I don't eat spicy strips B. 50g or less C. 50~100g D. 100~300g E. 300g and more.

9. Which kind of packages of spicy strips you often eat is?

A. Transparent bags.

B. Opaque bags.

C. Transparent and individual small package.

D. Opaque and individual small package.

10. What kind of spicy strip do you often eat?

A. Soggy B. Crisp C. Chewy

11. What is the shape of the spicy strips you often eat ?

A. Filamentous B. Rod-shaped C. Flaky D. Granular

12. What is the flavor of spicy strips you often eat?

A. Picante B. Sweet and spicy C. Numbing spicy D. Sweet and numbing E. Slightly sweet and spicy

13. How much money do you spend on spicy strips per month?

A. 0~30 yuan B. 30~50 yuan C. 50~100 yuan D. 100 yuan and more

14. What is your main consideration for choosing and purchasing spicy strips (multiple choice)?

A.Price B. Convenience for purchase C. Flavor D. Brand E. Production date / shelf life

F. No special considerations.

15.How do you buy spicy strips (multiple choice)?

A. Convenience stores and supermarkets B. School kiosks C. Mobile vendors D. Wholesale markets

Part 3. Related food consumption questionnaire

1. Frequency of soggy spicy strips consumption

A.Basically not eat B. 1-3 times a week C. 3-5 times a week D. More than 5 times a week

2. Frequency of crisp spicy strips consumption

A. Basically not eat B. 1-3 times a week C. 3-5 times a week D. More than 5 times a week

3. Frequency of chewy spicy strips consumption

A.Basically not eat B. 1-3 times a week C. 3-5 times a week D. More than 5 times a week

4. Frequency of electric grill food consumption

A.Basically not eat B. 1-3 times a week C. 3-5 times a week D. More than 5 times a week

5. Frequency of Charcoal grill food consumption

A.Basically not eat B. 1-3 times a week C. 3-5 times a week D. More than 5 times a week

6. Frequency of smoked food consumption

A.Basically not eat B. 1-3 times a week C. 3-5 times a week D. More than 5 times a week

7. Frequency of fried food consumption

A.Basically not eat B. 1-3 times a week C. 3-5 times a week D. More than 5 times a week

8. Frequency of cigarette consumption

A.Basically no smoking B. 1-3 times a week C. 3-5 times a week D. More than 5 times a week
